# Geographic abundance patterns explained by niche centrality hypothesis in two Chagas disease vectors in Latin America

**DOI:** 10.1371/journal.pone.0241710

**Published:** 2020-11-04

**Authors:** Mariano Altamiranda-Saavedra, Luis Osorio-Olvera, Carlos Yáñez-Arenas, Juan Carlos Marín-Ortiz, Gabriel Parra-Henao

**Affiliations:** 1 Centro de Investigación en Salud para el Trópico (CIST), Universidad Cooperativa de Colombia, Santa Marta, Colombia; 2 Politécnico Colombiano Jaime Isaza Cadavid, Medellín, Antioquia, Colombia; 3 Department of Ecology and Evolutionary Biology, University of Kansas, Lawrence, Kansas, United States of America; 4 Laboratorio de Ecología Geográfica, Unidad de Conservación de la Biodiversidad, UMDI-Sisal, Facultad de Ciencias, Universidad Nacional Autónoma de México, Mexico City, Mexico; 5 Departamento de Ciencias Agrarias, Universidad Nacional de Colombia, Facultad de Ciencias Agrarias, Medellín, Colombia; 6 National Health Institute (Instituto Nacional de Salud), Bogotá, Colombia; Federal University of Mato Grosso do Sul, BRAZIL

## Abstract

Ecoepidemiological scenarios for Chagas disease transmission are complex, so vector control measures to decrease human–vector contact and prevent infection transmission are difficult to implement in all geographic contexts. This study assessed the geographic abundance patterns of two vector species of Chagas disease: *Triatoma maculata* (Erichson, 1848) and *Rhodnius pallescens* (Barber, 1932) in Latin America. We modeled their potential distribution using the maximum entropy algorithm implemented in Maxent and calculated distances to their niche centroid by fitting a minimum-volume ellipsoid. In addition, to determine which method would accurately explain geographic abundance patterns, we compared the correlation between population abundance and the distance to the ecological niche centroid (DNC) and between population abundance and Maxent environmental suitability. The potential distribution estimated for *T*. *maculata* showed that environmental suitability covers a large area, from Panama to Northern Brazil. *R*. *pallescens* showed a more restricted potential distribution, with environmental suitability covering mostly the coastal zone of Costa Rica and some areas in Nicaragua, Honduras, Belize and the Yucatán Peninsula in Mexico, northern Colombia, Acre, and Rondônia states in Brazil, as well as a small region of the western Brazilian Amazon. We found a negative slope in the relationship between population abundance and the DNC in both species. *R*. *pallecens* has a more extensive potential latitudinal range than previously reported, and the distribution model for *T*. *maculata* corroborates previous studies. In addition, population abundance increases according to the niche centroid proximity, indicating that population abundance is limited by the set of scenopoetic variables at coarser scales (non-interactive variables) used to determine the ecological niche. These findings might be used by public health agencies in Latin America to implement actions and support programs for disease prevention and vector control, identifying areas in which to expand entomological surveillance and maintain chemical control, in order to decrease human–vector contact.

## Introduction

Studies on species abundance at different spatial and temporal scales provide insight into the community structure [1]. However, establishing general rules about geographic patterns in populations abundance is difficult [2, 3]. Researchers have proposed multiple hypotheses to describe the relationship between species distributions and geographic abundance patterns [4]. An old macroecology hypothesis states that species might be most abundant in the center of their geographic ranges, that is, the abundant-center hypothesis [5]. Some hypotheses state that abundance is explained not just the geographic position of populations along their distribution but also species’ environmental preferences. In this regard, some ecologists have evaluated the relationship between population density and distributional patterns using the outputs of ecological niche models (ENMs) as predictors of population abundance [6–9]. However, at geographical scales, the most recent abundant-niche-centroid hypothesis, states that abundance decreases as a function of the distance to the ecological niche centroid (DNC) [10]. Here, abundance decreases from optimal conditions at the centroid toward marginal conditions [11, 12]. Therefore, the relationship between abundance and the DNC is expected to be negative [13]. On the basis of the conjecture that demographic parameters and the location of populations in niche space are related [13–16].

Learning about the relationship between population abundance and DNC has direct repercussions on some of the most relevant environmental challenges (such as biological invasions, habitat conservation, climate change, emerging diseases, and food security) [17]. Thus, the need for evaluating this relationship has become yet more pressing. In some cases, when abundance or density data are available, abundance can be modeled directly using Poisson regression or other statistical methods that use environmental predictors [18]. However, obtaining data on abundance is complicated and demanding, especially for rare species [19]. Therefore, ENMs is a low-cost option to model abundance in different spatial scales [20].

The evidence of a positive relationship between species abundance and environmental suitability as a general pattern in nature is controversial [21]. The use of ENMs to assess the geographic distribution of population abundance is relatively recent, starting in the early twenty-first century [4, 22]. Nielsen et al [7] analyzed the relationship between occurrence and abundance of two species with different backgrounds (the bracken fern *Pteridium aquilinum* (Kuhn in Kersten, 1879) and the moose *Alces alces* (Linnaeus, 1758)) and concluded that environmental factors affecting population abundance might differ from those limiting population distribution. In this regard, several studies have investigated relationships between species distribution and geographic abundance patterns by using ENM and DNC approaches [15, 22–24]. Braz et al [21] reported the ability of distinct modeling methods to predict species abundance and recommended that the relationship between population abundance and environmental suitability be carefully interpreted when using ENMs to predict species distribution, because biotic interactions can be the main driver of local population abundance within highly suitable environments. In addition, the predicted abundance niche distance relationship is not common [5, 11], but the differences between findings can instead rather be explained by methodological issues [25].

The characterization of the ecological fundamental niche is crucial to test the abundant niche-centroid hypothesis, which would allow the estimated centroid that truly represents its environmental optimum [13]. On the basis of the assumptions made about the species geographic distributions, we can interpret the environmental suitability estimated by climate-only models as an approximation of the fundamental niche [21]. A starting point to study the geographic variation of population abundances is the biotic, abiotic, movement (BAM) framework, which states that species distribution depends on three factors: dynamically linked biotic interactors (**B**), unlinked abiotic stressors (**A**), and dispersal capacity (**M**). The area where all three of these conditions meet represents species’ actual distribution and its occupied niche [26]. Using BAM components and scenarios allows us to exemplify situations in which the DNC estimated from distributional might or might explain species abundance [27]. Specifically, when using correlative models, BAM configurations in which species are not in climatic equilibrium would lead to underestimation of the fundamental niche biasing the characterization of the true centroid.

Information about the spatial population distribution patterns of insect vectors might explain their behavioral traits and the effects of environmental factors on the population [28]. Insect abundance and distribution are regulated by several biotic and abiotic factors and their interactions [29–32]. For example, precipitation, temperature and humidity are the most important elements restricting abundance and regulating insect communities [33–35]. Therefore, learning about the mechanisms underlying the incidence of vector-borne diseases because of environmental changes will allow us to plan control strategies at different spatial scales [36].

Ecoepidemiological scenarios for the transmission of Chagas disease are complex, so measures for vector control to decrease human–vector contact and prevent infection transmission are difficult to implement in all geographic contexts [37]. In addition, because of the ecological, geographic, and demographic heterogeneity of Chagas disease, more and better tools are required for the proper characterization of its risk and transmission scenarios [38]. Thus, secretaries of health or ministries of health in Latin America might use the relationship between triatominae species’ distribution and geographic abundance patterns to support disease prevention and vector control programs.

This study tested the abundant- niche centroid hypothesis to (i) determine whether the tendency is toward negative relationship between population abundance and the DNC; (ii) estimate the correlation between population abundance and Maxent environmental suitability,; and (iii) map, at a fine spatial scale, the risk of Chagas transmission using, as input, the model that better explains geographic abundance patterns of two vector species of Chagas: *Triatoma maculata* (Erichson, 1848) and *Rhodnius pallescens* (Barber, 1932) in Latin America. Our null hypothesis is that the internal structure of the niche explains the abundance patterns of the species.

## Material and methods

### Study area and input data

The area of study extended from northern Mexico to the austral ends of Chile and Argentina. We selected two triatomine species that are secondary Chagas disease vectors in Latin America (*T*. *maculata* and *R*. *pallescens*). We compiled occurrence records from multiple sources, including the available literature, online data on occurrence records from the Global Biodiversity Information Facility (http://www.gbif.org/; accessed on August 17, 2019), and a database from some colleagues and field observations from our long-term studies (see the summary in S1 Table). We also used Moran’s I coefficient and semivariogram graphs to eliminate spatial autocorrelation. In addition, we downloaded abundance data from the literature included in the database between 1971 and 2019 (PubMed, Scielo, ScienceDirect, Web of Science, Google Scholar, JSTOR, and Directory of Open Access Journals). The spatial information was validated using the Leaflet library in R software [39], which verified that each point corresponding to each record was correctly located according to the reported location, following the point-to-radio method. This method ignores the fact that a locality record always describes an area, not a dimensionless point, and that collecting might occur anywhere within the area denoted, providing only a point for a georeferenced record [40].

We used the 19 bioclimatic variables from CHELSA v1.1 online database as environmental data [41]. These variables were built on the basis of monthly averages of climate data, (mainly temperature and precipitation), as collected from meteorological stations, for 1979–2013, and interpolated to the global surface [41], with a spatial resolution of 30 arc-seconds (~1 km^2^ cell size). We conducted an initial correlation analysis to avoid collinearity related issues although the ENM methods used here have proved to be robust when such issues appear [42, 43]), and to increase computational speed. In other words, using R software, we removed from the analysis one from each pair of environmental variables, for which Pearson product–moment correlations were >0.8 [44]. In addition, on the basis of the variable contribution estimates generated by the jackknife plot in the Maxent output and correlation coefficients, we determined which variables to retain for further evaluation [45]. We obtained three sets of bioclimatic variables per species, which we used to build niche models (Table 1). To identify a calibration area (**M**) per species, we considered the global terrestrial ecoregions of the world [46], with at least one presence record of the species in question as accessible regions. Region **M** represents the areas to which a species has had access over a relevant period and has, therefore “tested” the associated environmental conditions for suitability [47, 48].

**Table 1:** Performance metrics of the selected model.

Species	Occurrence records	Model settings	Set of variables	*p*-value-(partial ROC)	Omission rate (<5%)	Delta AICc	Parameters
*Rhodnius pallescens*	228	RM = 0.9; FC = lp; Set 3	Bio1, Bio5, Bio7, Bio8, Bio12, Bio18	0	0.049	0	11
*Triatoma maculata*	271	RM = 0.3; FC = lq; Set 1	Bio1, Bio3, Bio8, Bio9, Bio10, Bio12, Bio19	0	0.067	0	11

RM, regularization multiplier; FC, feature classes (l = linear, q = quadratic, p = product) and sets of environmental variables per each species; ROC, receiver operating characteristic; AIC, Akaike information criterion.

Bio1 = annual mean temperature; Bio3 = isothermality; Bio5 = maximum temperature of the warmest month; Bio7 = annual temperature range; Bio8 = mean temperature of the wettest quarter; Bio9 = mean temperature of the driest quarter; Bio10 = mean temperature of the warmest quarter; Bio18 = precipitation of the warmest quarter; Bio19 = precipitation of the coldest quarter.

### Ecological niche modeling

Each calibration process involved creating and evaluating candidate models using Maxent 3.4.1 [49]. We explored the best model parameterization using the R package kuenm [50] based on distinct parameter settings: 3 sets of environmental variables, 17 values of regularization multipliers (0.1, 0.2, 0.3, 0.4, 0.5, 0.6, 0.7, 0.8, 0.9, 1, 2, 3, 4, 5, 6, 8, 10) and 7 potential combinations of three feature classes (linear [L], quadratic [Q], product [P], linear + quadratic [LQ], linear + product [LP], quadratic + product [QP], linear + quadratic + product [LQP]). We tested 357 candidate models for each species. We selected model performance and best candidate models first by significance, second by performance, and subsequently by the Akaike information criterion (AIC) AICCc, delta AICCc, weight AICCc and predictive power (omission rate, E = 5%) [51]. We generated the final model and its evaluation by bootstrap for each species using 10 replicates with raw outputs, and these were projected to the entire study area (Latin America) [50]. In addition, we established a threshold to convert raw Maxent outputs into binary maps of suitable versus unsuitable environments using the reclassification threshold of lower training presence (LTPT) [52] under an allowable error rate of E = 5%. The thresholds were 0.0011 for *T*. *maculata* and 0.00015 for *R*. *pallescens*.

### Geographic variation in population abundance

We got a total of 407 abundance data, including *T*. *maculata* (n = 197) and *R*. *pallescens* (n = 210). We measured distances between observations of population abundances (S2 Table) and the niche centroid. To estimate the niche and its centroid, we used the minimum-volume ellipsoid (MVE) approach with the *ellipsoid_selection* function of the R package ntbox [53]. The *ellipsoid_selection* function has a model calibration and selection protocol that allows us to select niche models that are statistically significant and have good performance. Next, we used the selected MVEs to fit models that related DNCs to abundance data.

First, we used the *ellipsoid_selection* function to build MVEs for all combinations of *n* environmental variables taken by *m*. Here, we estimated each model using the *cov_center* method, which calls the MVE algorithm of the *cov*.*rob* function available in the R package MASS [54]. For each environmental combination, the MVE algorithm builds an ellipsoid of the smallest volume that contains a *k* proportion of training points [55]. We estimated the statistical significance of models via receiver operating characteristic (ROC) test for testing data [56] and calculated the performance as omission rates for both training and testing records (via the *inEllipsoid* function of ntbox). The algorithm selected models that had a *p*-value of the partial ROC test of ≤ 0.05 and an omission rate of ≤ 0.05; the proportion of training points inside the ellipsoid was *k* = 0.95, and the environmental predictors used to fit the MVEs were bioclimatic variables (CHELSA) that manifested correlations of *p* ≤0.8. We fit 5017 candidate models for each species, in addition to the MVEs generated for all possible combinations of 2 or 3 variables selected from among 19 bioclimatic variables.

Second, we computed the Mahalanobis DNCs and environmental values of population abundance records (note that these records are independent of the training and testing occurrences used in the ENM part) using the niche centroid and minimum-volume covariance matrix of the selected MVEs. We created a matrix with the environmental information about population abundance records and computed each row’s DNCs with respect to the minimum-volume covariance matrix using the *mahalanobis* function in R.

Third, we calculated Spearman correlations between the DNC and population abundance using the *cor*.*test* function in R [17]. To evaluate which ENM method provides a better explanation of the geographic abundance patterns, we estimated the correlation between outputs of the Maxent models selected by kuenm and population abundance and then compared it with that concerning the niche centroid–based distances. Finally, we applied the ellipsoid_fit function of ntbox to build a suitability map based on the information about the MVE that provided the best fit to population abundance data. We reclassified this map into four transmission risk categories by dividing its values into four classes representing suitability quartiles. To introduce the BAM diagram approach, we cut the risk maps cut using the **M** layer for each species.

## Results

### Ecological niche modeling

**Fig 1:**
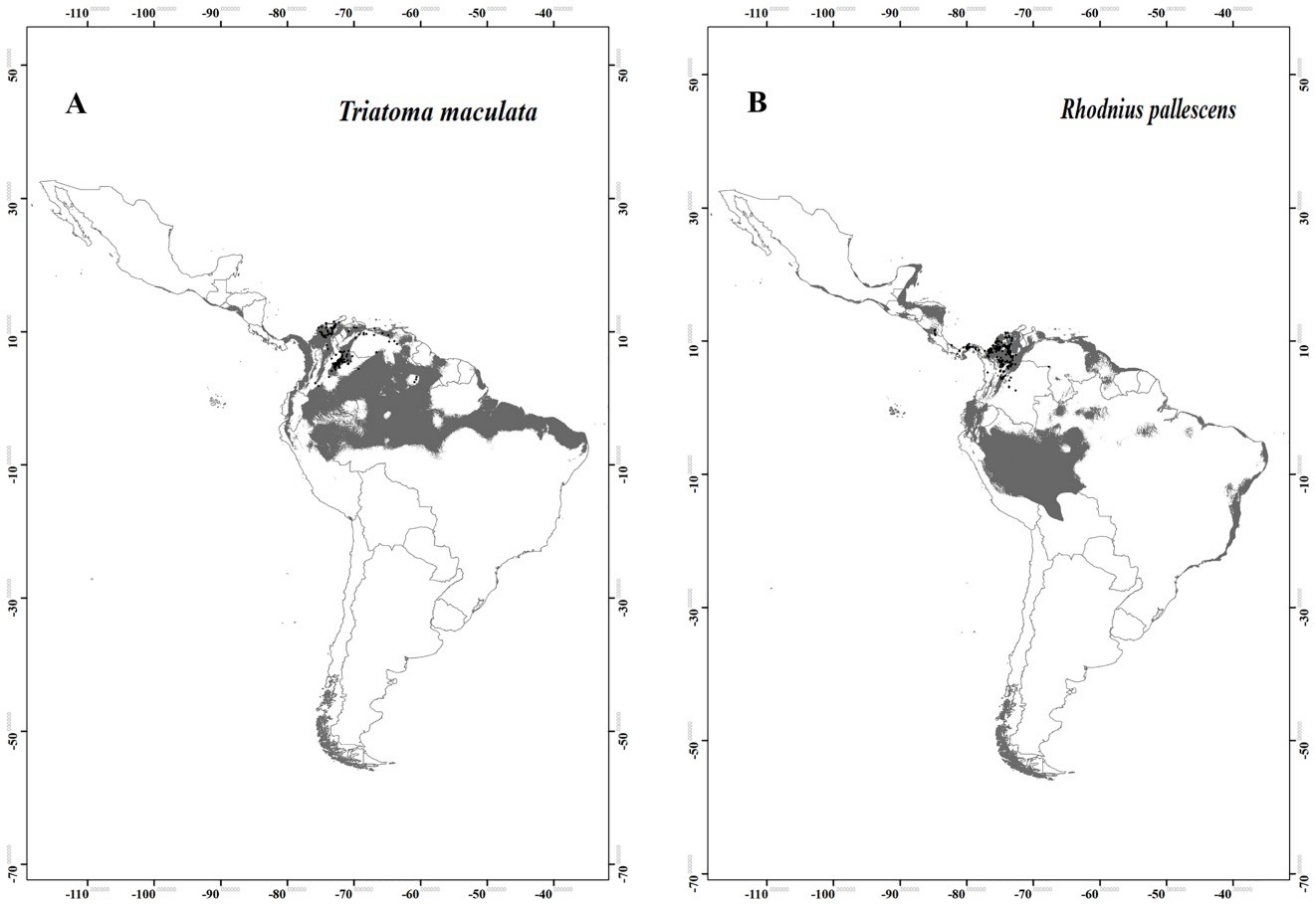
Potential distribution maps for (A) *Triatoma maculata* and (B) *Rhodnius pallescens*- Models were calibrated across the hypothesized area of dispersion (M) and transferred across all Latin America. Black dots are indicated occurrences; gray areas modeled suitable conditions; and white areas unsuitable conditions.

The complete occurrence database included 499 records of the presence of species, including *T*. *maculata* (n = 271) and *R*. *pallescens* (n = 228) [17]. The final models were highly predictive of species distribution (Table 1). The potential distribution binary models estimated for *T*. *maculata* indicated that the area with the highest environmental suitability extended from Panama to northern Brazil (Fig 1A). *R*. *pallescens* showed a more restricted potential distribution, with environmental suitability mainly in the coastal zone of Costa Rica, Nicaragua, Honduras, Belize and the Yucatán Peninsula in Mexico, northern Colombia, Acre and Rondônia States in Brazil and a small portion west of the Brazilian Amazon (Fig 1B). Environmental variables with a larger contribution to the *T*. *maculata* model, were the annual mean temperature, isothermality, and precipitation of the coldest quarter; while those with a larger contribution to the *R*. *pallescens* model were the temperature annual range, annual precipitation, and precipitation of the warmest quarter.

### Geographic variation in population abundance

**Fig 2:**
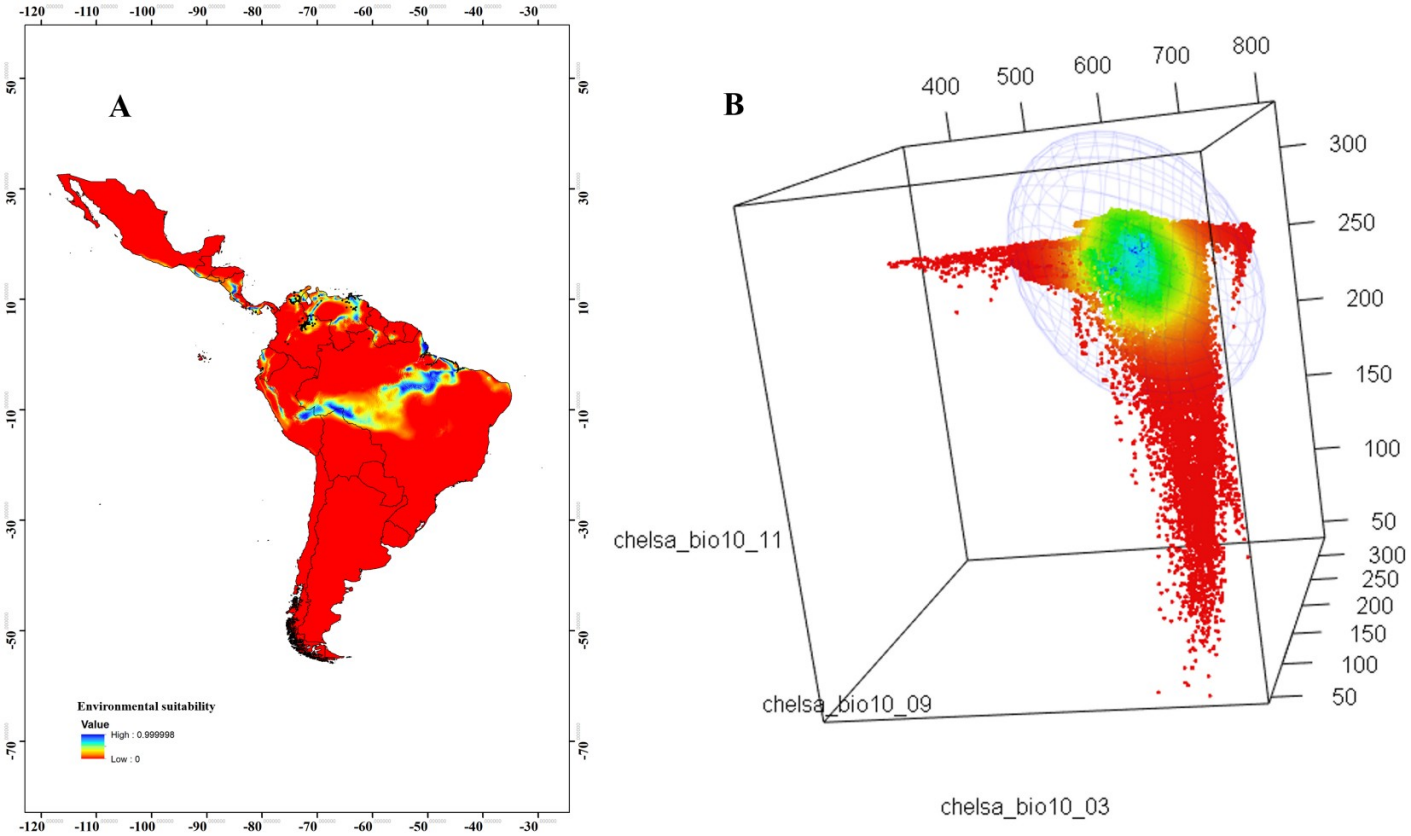
Geographical areas closer to the niche centroid. (A) Environmental suitability model of *Triatoma maculata* representing the DNC, with values from 0 (blue) to 1 (red); population abundance records are represented in black. (B) Environmental space (MVE). DNC, distance to the niche centroid; MVE, minimum-volume ellipsoid. Bio3 = isothermality; Bio9 = mean temperature of the driest quarter; Bio11 = mean temperature of the coldest quarter.

**Fig 3:**
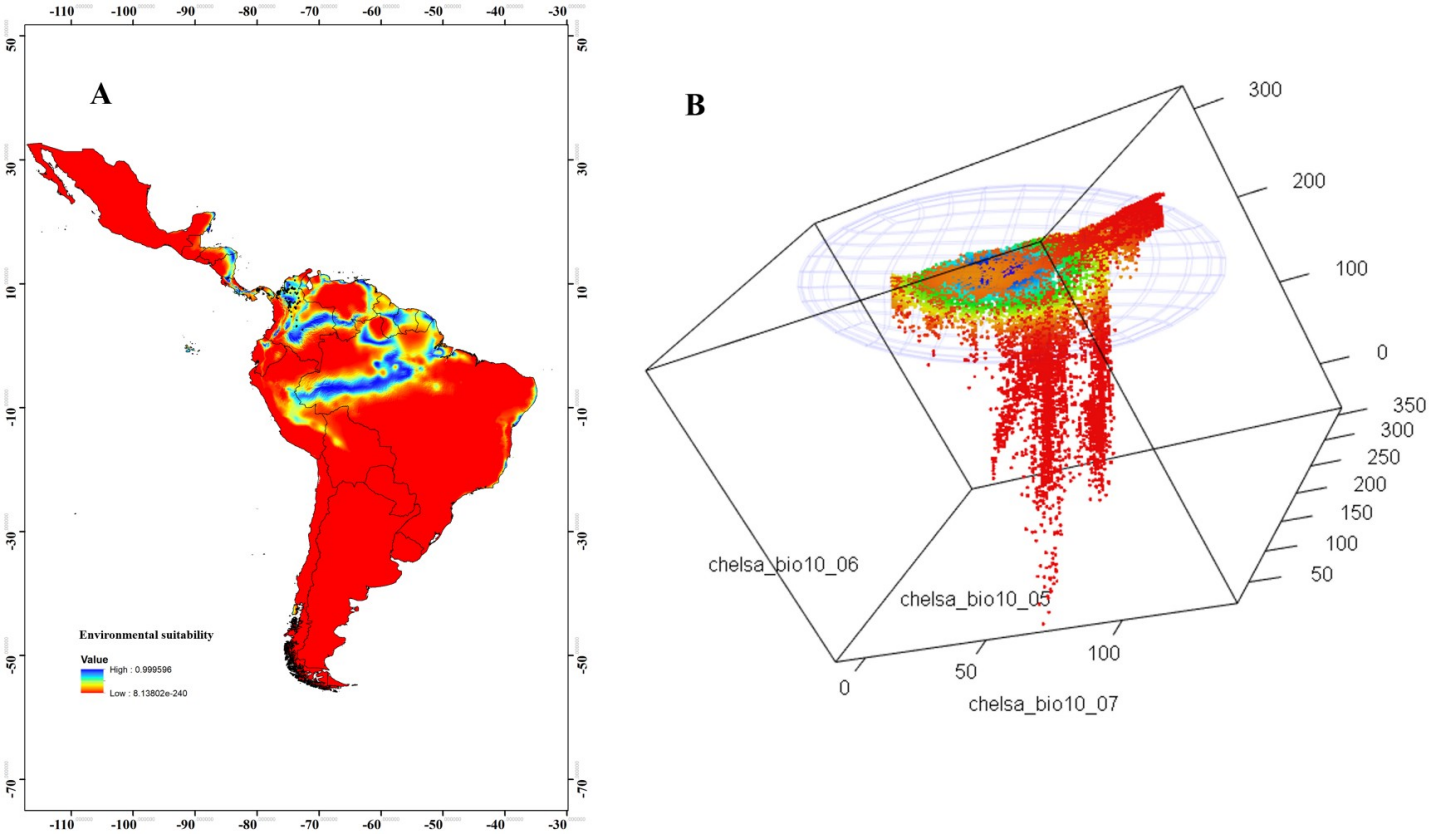
Geographical areas closer to the niche centroid. (A) Environmental suitability model of *Rhodnius pallescens*, representing the DNC, with values from 0 (blue) to 1 (red), abundance records are represented in black. (B) Environmental space (MVE). DNC, distance to the niche centroid; MVE, minimum-volume ellipsoid. Bio5 = maximum temperature of the warmest month; Bio6 = minimum temperature of the coldest month; Bio7 = annual temperature range.

We selected an MVE model per species (Table 2). The geographical representation of environmental suitability obtained from DNC showed geographical areas closer to the niche centroid, indicated that these are the places where more abundant populations are expected. For *T*. *maculata*, in Colombia, these areas were mostly located in the Caribbean and Andean natural regions, much of northern Venezuela, central Brazil, and small areas in Peru, Ecuador, and Central America (Fig 2A). The niche space plot showed that the majority of environmental conditions in the study area were far from the niche centroid (Fig 2B). For *R*. *pallescens*, the geographical areas closer to the niche centroid had a similar pattern as *T*. *maculata*; areas closer to the niche centroid were in Guyana, Suriname, French Guiana, the Northern Caribbean coast, and eastern plains in Colombia (Fig 3A). The environmental background was closer to the niche centroid (Fig 3B).

**Table 2:** Performance metrics of the selected MVE model.

Species	Number of variables	Set of variables	Training	Testing occurrence Records	*p*-Value	R^2^
*Rhodnius pallescens*	4	Bio1, Bio5, Bio6, Bio7	135	167	0.00084	0.31
*Triatoma maculata*	3	Bio3, Bio9, Bio11	222	340	0.00057	0.11

MVE, minimum-volume ellipsoid.

Bio1 = annual mean temperature; Bio3 = isothermality; Bio5 = maximum temperature of the warmest month; Bio6 = minimum temperature of coldest the month; Bio7 = annual temperature range; Bio9 = mean temperature of the driest quarter; Bio11 = mean temperature of the coldest quarter.

**Fig 4:**
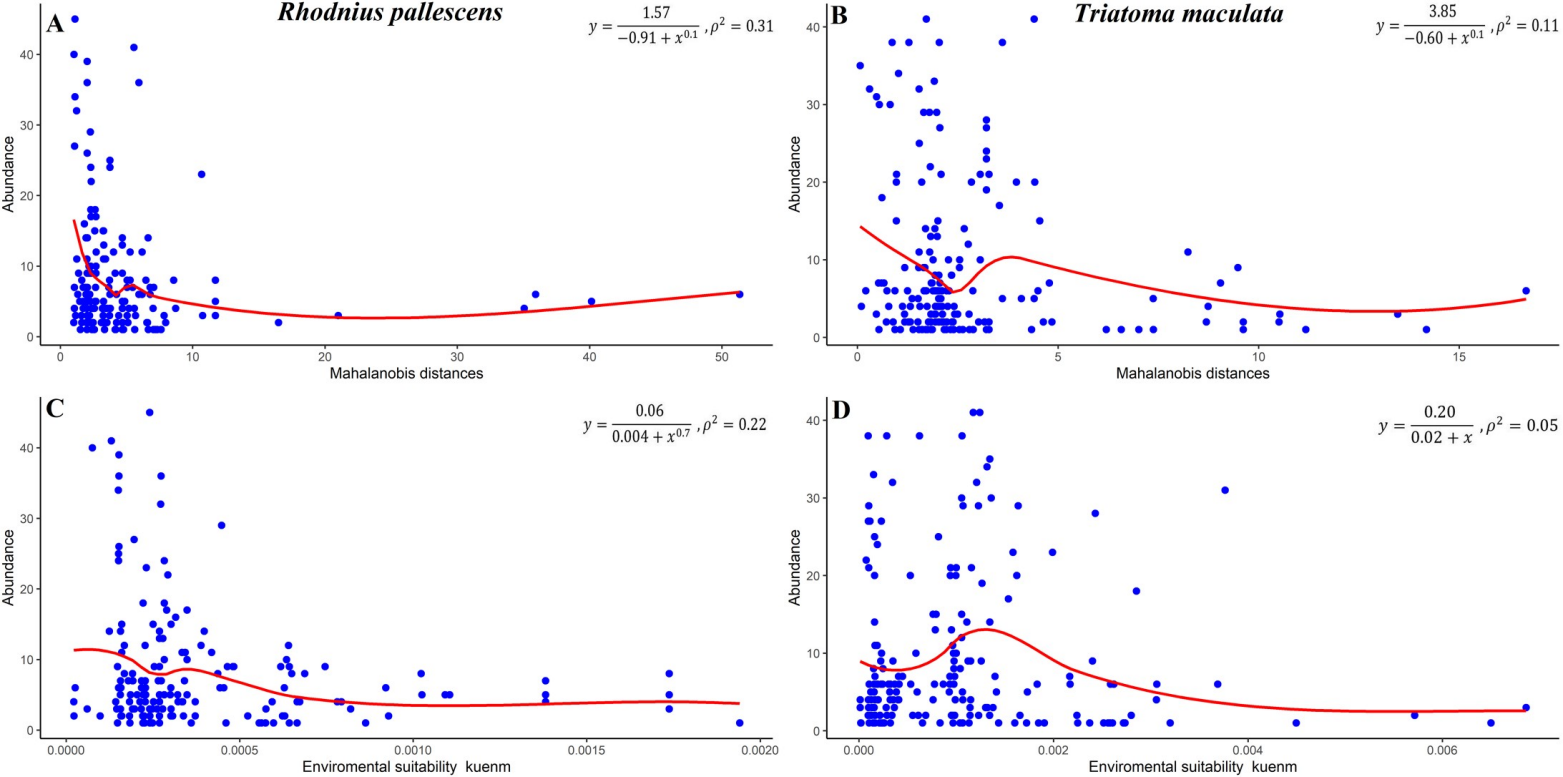
Population abundance–DNC relationships based on MVEs and Mahalanobis distances. Population abundance was low far from the niche centroid in (A) *Triatoma maculata* and (B) *Rhodnius pallescens* (based on Maxent environmental suitability). There was no significant relationship between environmental suitability and vector abundance in (C) *Triatoma maculata* and (D) *Rhodnius pallescens*. DNC, distance to the niche centroid; MVE, minimum-volume ellipsoid.

The negative slope of the relationship between population abundance and the DNC in both species indicated that the local population abundance is low far from the niche centroid and that this effect is stronger at the upper limit of the abundance distribution (Fig 4A and 4B). There was no significant relationship between environmental suitability and vector abundance (Fig 4C and 4D).

**Fig 5:**
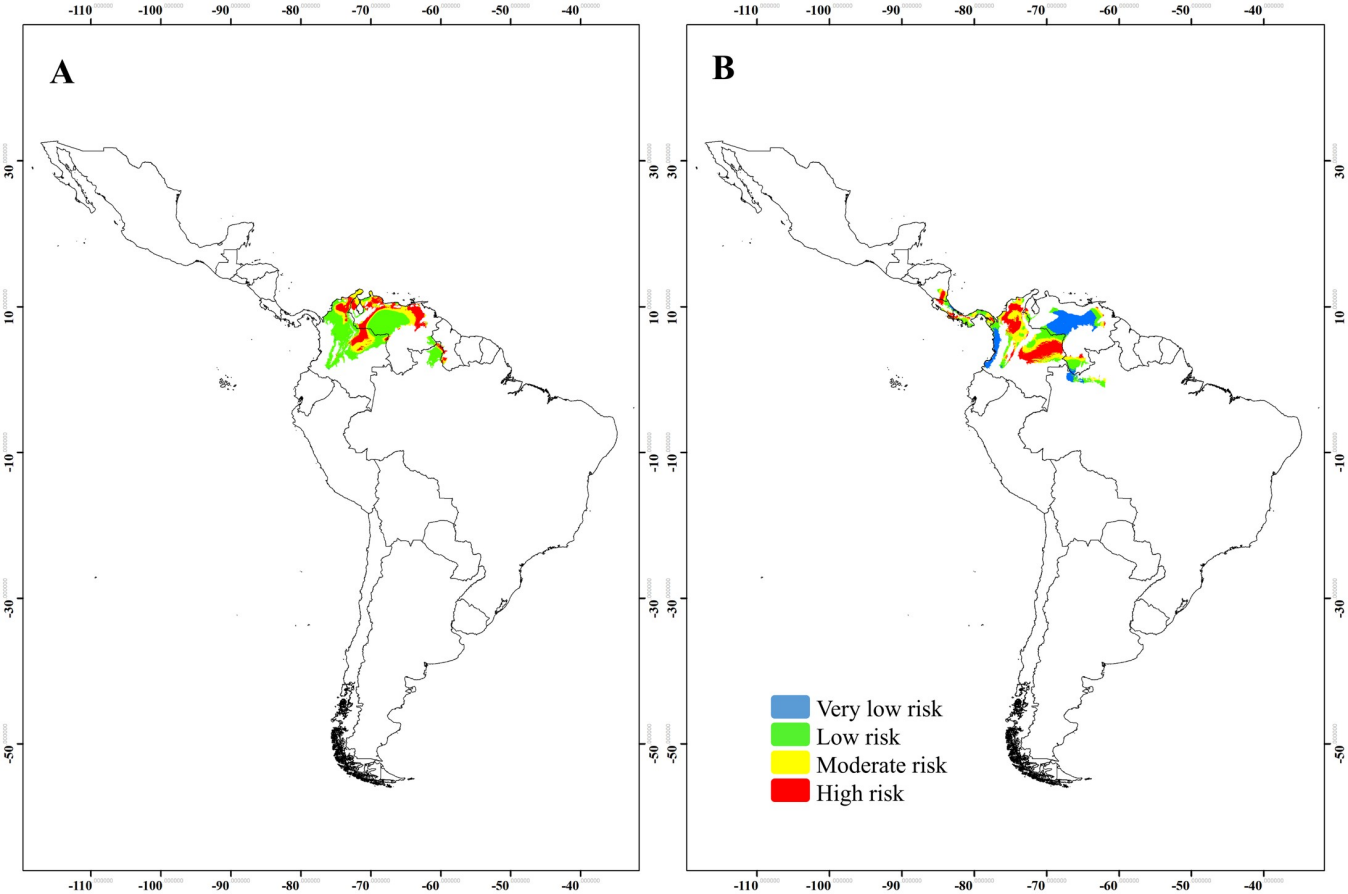
Map of transmission risks categories obtained from the environmental suitability model of the DNC. (A) *Triatoma maculata* and (B) *Rhodnius pallescens*. DNC, distance to the niche centroid.

The spatial representation of transmission risks for *T*. *maculata* showed that t northern of Colombia and Venezuela, and in the central-west Brazil, are high-transmission-risk areas, while the northernmost and southernmost areas of Latin America have low transmission risk. For *R*. *pallescens*, the high-and moderate-risk areas, were in much of Colombia, Venezuela, northern Brazil and the Guiana region (Fig 5).

## Discussion

Ecological niche modeling of insects with medical relevance are a useful tool for designing vector control measures and offering base information to understand the ecoepidemiological aspects of diseases [57–59]. *R*. *pallecens* has a more extensive, potential latitudinal range compared to that previously reported in the literature, with more suitable areas in Costa Rica, Panama, and Colombia [60–63], indicating that this species has a high probability of colonizing new habitats, increasing its potential distribution to the south of the continent [63]. The potential distribution increases the risk of *Trypanosoma cruzi* (Chagas, 1909) transmission in regions where it is either absent or reported occasionally, such as Ecuador, eastern Peru, and Bolivia [64]. In addition, the *T*. *maculata* distribution model corresponds with previous records, mainly in Colombia, Venezuela, and northern Brazil [65–68]. However, the prediction of environmentally suitable areas for the occurrence of this species in Panama, where it has not been previously reported [68].

The metrics used to validate our calibration model (partial ROC, omission rate, and delta AICc) suggest that the predictions are reliable. According to our calibration model’s predictions, there is little probability of co-occurrence of the species for settlement in areas with favorable environmental conditions; some places with potential sympatry are Panama, the Colombian Caribbean, and northern Venezuela. Geographic co-occurrence implies biotic interactions, such as vectoring and hosting pathogens. This proposition is a promising topic in epidemiology and public health that can be examined by taking a co-occurrence networks approach [69].

The effects of climate on triatomines have been studied, in details, especially highlighting a few temperature based variables as factors that affect their distribution [70, 71]. Our results partially corroborate those obtained by De La Vega and Chilman [72], who reported isothermality as one of the environmental variables with the most significant contribution in models of six triatomine species, and minimum temperature of the coldest month as the limiting factor for the distribution of most species evaluated in this study. Although temperature (25°C–58°C) and relative humidity (~ 70%) are variables with a decisive impact on triatomine distribution [73], this study found that some variables derived from rainfall have a significant contribution in the potential distribution models of the species studied (such as precipitation of the coldest quarter for *T*. *maculata* and annual precipitation and precipitation of the warmest quarter for *R*. *pallescens*). These findings are consistent with other studies [74, 75]. Precisely, precipitation of the driest month is a variable with the highest contribution for *Triatoma dimidiata* (Latreille, 1811) [74], the seasonality of precipitation and the same precipitation of the driest month are more critical for *Triatoma pallidipennis* (Stal, 1872) [75].

The abundant niche centroid hypothesis is a current topic in biogeography and ecology [17]. However, more studies are required in order to assess the scope of this approach [5, 11, 17]. This is the first study to evaluate abundant niche centroid hypothesis using insects with high ecoepidemiological importance as a study model. We believe that under BAM configurations in insect vectors of human disease such as Triatominae, the effect of **B** (biotic) over **Gp** (potential distribution area) and the geographic abundance patterns is strong, mainly because of the relevance of the interaction with humans (hematophagous behavior) and interspecific interactions [76, 77]. However, the population abundance–DNC relationships at large spatial scales are negative, where population abundance increases with the proximity to the niche centroid, indicating that population abundance is limited by the set of scenopoetic variables at coarser scales used to determine the ecological niche. This pattern is known as the Eltonian noise hypothesis [78].

In contrast, some studies have assessed the capacity of logistic Maxent environmental suitability to explain population abundance patterns, using the default software parametrization, and showed no significant relationship between environmental suitability and spatial abundance patterns [6, 22, 23]. Our results are consistent with these previous findings, but our study was performed under conditions different from previous ones. To our knowledge, this is the first study to use the R package kuenm to test these relationships. The R package kuenm is based on a robust model calibration process, facilitating the creation of final models based on model significance, performance, and simplicity [50].

The geographical areas where we expect more abundant *T*. *maculata* and *R*. *pallescens* populations, according to the spatial representation of the niche centroid, are terrestrial habitats such as the Amazon rainforest, tropical dry forests, and tropical and subtropical grasslands [46]. Changes in land use and land cover alter the exchange of heat, moisture, momentum, trace-gas fluxes, and the climate at a local scale [79]. These anthropogenic activities could favor the abundance of the species evaluated, because for *R*. *pallescens*, the land use transformation, generalized in its distribution area, could induce changes in the vector ecology, initiating a gradient that leads to synanthropic behavior [80]. *R*. *pallescens* has been found under both domestic and sylvatic conditions. Colonies are also found in sylvatic ecotopes, such as the crowns of at least four palms species: *Attalea butyracea* (Mutis ex L.f.) Wess.Boer, *Cocos nucifera* L., *Oenocarpus bataua* (Burret, 1929), and *Elaeis oleifera* (Cortés, 1897) [60, 81]. In addition, and high densities of *A*. *butyracea* in forests are commonly associated with anthropogenic activities, such as hunting of seed predators of these palms and past agricultural activity [82]. *Attalea butyracea’s* presence and abundance are the main components of the habitat defining the ecological niche of *R*. *pallescens* [62, 80, 83]. In contrast, the differential relevance of *T*. *maculata* in transmission cycles from distinct geographical areas indicates that the species can quickly adapt to stable artificial ecotopes its their natural habitats are destroyed, and its distinct ecological behaviors have different epidemiological implications [67, 83]. Further validation of our results via field investigations to identify present species is required.

One of the main aspects to be considered when ascertaining the relationship between niche centrality and population abundance is that dispersal between populations should be limited [15, 17]. This assertion is consistent with our results, because the natural dispersion capacity of triatomine vectors is not wide [84, 85]. For example, *Triatoma infestans* (Klug in Meyen, 1834) register an effective flight range of at least 200 m [86]. *Rhodnius* bugs residing in *A*. *butyracea* can invade domestic environments from within a circumference of at least 100 m [87]. In general, migration in urban and peri-urban areas is influenced by factors such as light bulbs and in forest habitats by the sylvatic–domestic zone distance [88].

Ecological niche modeling results for spatial epidemiology are widely used to generate risk maps and answer ecological and distributional questions related to the complex disease system [89, 90]. For example, recently, a risk map of cutaneous leishmaniasis based on anthropogenic, climatic and environmental factors was designed [91]. Venezuela, northern Colombia, northern and central-west Brazil, and the Guiana region are potential at-risk areas for Chagas disease. This result matches zones with current disease transmission [92], except for Guyana, Surinam, and French Guyana, where Chagas disease is not a public health concern [92, 93], although *T*. *infestans* is the main vector in Brazil [94] and *Rhodnius prolixus* (Stal, 1859) in Colombia and Venezuela [95, 96]. In 2011, all the previously endemic Central America countries were formally certified as free of Chagas disease transmission thanks to control strategies for eradication of the main domestic vector, *R*. *prolixus* [97]. Some South American countries were certified, too [98]. *R*. *pallescens* and *T*. *maculata* have vectorial relevance in some South and Central America countries and are considered a potential concern in ecoepidemiologically as candidates to replace the domestic *R*. *prolixus* once it is eliminated from homes by control campaigns [81].

Several other important factors drive the epidemiological risk due to the heterogeneous distribution of Chagas disease in Latin America [99]. Therefore, future models must consider sociodemographic factors, reservoir distribution, human migration, palm distribution, level of human action in nature, and housing materials [100, 101]. This study had a few limitations. First, we did not evaluate sociodemographic factors because of a lack of data and, in some cases, a lack of methodological tools that allow incorporation in ENMs [26]. Second was the availability of robust population abundance data based on routine sampling and temporal divergence between population abundance data and climate. However, ours is a novel approach that uses as input one of the most important risk factors (potential vector abundance) and might be useful to establish or implement control measures at a regional level, as well as alert health systems and authorities in areas with higher risk of disease transmission.

Understanding how geographic abundance patterns of some insects increase the human risk of exposure to vector-borne disease agents and evaluating the effects of differences among species (e.g., differences in dispersal capacity) on population abundance patterns and geographic distribution patterns are critical for targeting limited prevention, surveillance, and control resources. This information will help public health entities efficiently direct surveillance and vector control interventions and will allow optimization of resources allocated for disease control by, for example, targeting places to monitor vector abundance, drug administration, or prevention education campaigns and identifying areas for the most effective use of pesticides [102]. However, public health research and policy have the challenge of incorporating the ecological dimension into management and vector control strategies to any important degree.

We conclude that, population abundances increase according to the proximity to the centroid, indicating that abundance is limited by the set of current scenopoetic variables at coarser scales (non-interactive variables) used to determine the ecological niche. Nevertheless, this relationship may be affected by different factors, including the variation of environmental conditions under the effect of the climate change. Thus, to assess how the population dynamic of *R*. *pallescens* and *T*. *maculata* respond to climate change using this methodological approach is an unexplored but important avenue for future investigation [17].

## Supporting information

S1 Table Occurrence records of Chagas disease vectors using ecological niche models. (XLSX)

S2 Table Population abundance data of Chagas disease vectors using MVE models. (XLSX)
